# Signal detection and analysis of adverse events associated with Genvoya^®^ based the FAERS database

**DOI:** 10.3389/fphar.2024.1439781

**Published:** 2024-12-04

**Authors:** Chengliang Wang, Yan Zhang, Xiting Tang, Guoping Zhang, Li Chen

**Affiliations:** ^1^ People’s Hospital of Ganzi Tibetan Autonomous Prefecture, Kangding, Sichuan, China; ^2^ Department of Pharmacy, West China Second University Hospital, Sichuan University, Chengdu, Sichuan, China; ^3^ Chinese Evidence-Based Medicine Center, West China Hospital, Sichuan University, Chengdu, Sichuan, China; ^4^ Department of Pharmacology, Faculty of Medicine and Nursing, University of the Basque Country, Leioa, Spain

**Keywords:** Genvoya^®^, adverse events, FAERS database, signal mining, HIV treatment

## Abstract

**Objective:**

This study aims to evaluate and understand the safety profile of Genvoya^®^ by mining and analyzing adverse drug event (ADE, adverse drug event) reports from the FDA Adverse Event Reporting System (FAERS, FDA Adverse Event Reporting System) database, thus providing valuable reference information for individuals infected with HIV.

**Methods:**

Data were obtained from the FAERS database, covering the period from the first quarter of 2015 to the fourth quarter of 2023, focusing on reports where Genvoya^®^ was the primary suspected drug. Data import and extraction were conducted using MySQL 8.0, with adverse events standardized according to the Medical Dictionary for Regulatory Activities (MedDRA, Medical Dictionary for Regulatory Activities) 27.0 terminology. Potential adverse event signals were identified through disproportionality analysis, including the reporting odds ratio (ROR, reporting odds ratio) method and the comprehensive standard by the Medicines and Healthcare products Regulatory Agency (MHRA, Medicines and Healthcare products Regulatory Agency) method. Statistical analyses and graphical representations were performed.

**Results:**

A total of 2, 376 adverse drug event reports related to Genvoya^®^ were analyzed. Reports from male patients accounted for 74.33%, while those from female patients accounted for 22.39%. Common adverse events included weight gain, drug interactions, and increased viral load. Additionally, new potential adverse reactions, such as fat redistribution, HIV-associated neurocognitive disorders, and meningoencephalitis, were identified. These reactions were not adequately described in the existing literature and drug labels.

**Conclusion:**

Multiple adverse reactions were observed with the use of Genvoya^®^. Clinicians should closely monitor these reactions and implement necessary preventive and intervention measures based on patient-specific conditions and treatment guidelines. Although this study has limitations, the analysis of FAERS database data has revealed various potential risks associated with Genvoya^®^, providing important safety references for HIV treatment.

## 1 Introduction

Antiretroviral therapy (ART) has significantly improved the quality of life and life expectancy of individuals infected with the human immunodeficiency virus (HIV). Within this context, the introduction of Genvoya^®^ offers an innovative treatment option. Genvoya^®^, a fixed-dose combination drug (FDC), comprises four active ingredients: 150 mg of Elvitegravir (EVG), an integrase inhibitor for HIV treatment; 150 mg of Cobicistat (COBI), a pharmacokinetic enhancer to boost EVG plasma levels; and Emtricitabine (FTC) and Tenofovir Alafenamide (TAF), which are nucleoside reverse transcriptase inhibitors (NRTIs) that inhibit viral replication. This medication has demonstrated high viral suppression rates in multiple international clinical studies (Angione et al., 2018), while its unique composition has shown reduced renal and bone toxicity (Eron et al., 2019; Arribas et al., 2016). Despite these advantages, the widespread use of Genvoya^®^ globally necessitates a thorough examination of its long-term safety, particularly regarding potential adverse drug events (ADEs) in various populations (Gantner et al., 2019; Wohl et al., 2016; DeJesus et al., 2012).

The FDA Adverse Event Reporting System (FAERS) is a crucial database for monitoring global drug safety. Analyzing FAERS data can uncover potential safety issues associated with Genvoya^®^ (Viswam et al., 2019). Although clinical trials provide initial evidence of Genvoya^®^'s safety and efficacy, analyzing real-world data can help identify ADEs not observed during clinical trials. This analysis is essential for offering a comprehensive safety assessment for the long-term treatment of HIV-infected individuals, thereby significantly enhancing their quality of life and treatment satisfaction (Maggiolo et al., 2016; Gallant et al., 2016). This study aims to evaluate and understand the safety of Genvoya^®^ by mining and analyzing adverse event reports from the FAERS database, providing valuable reference information for HIV-infected individuals.

## 2 Materials and methods

### 2.1 Data source

The data utilized in this study were sourced from the FDA Adverse Event Reporting System (FAERS) database, which is updated quarterly. This extensive database collects post-marketing adverse event reports, including details on the number of reports, patient age, gender, and the severity of adverse drug events (ADEs). It comprises seven main tables: patient demographic and administrative information (DEMO), drug information (DRUG), adverse drug reaction information (REAC), patient outcome information (OUTC), information on report sources (RPSR), drug therapy start and end dates (THER),indications for use/diagnosis (INDI), and deleted cases (DELETED).

### 2.2 Data processing

The database was searched using both generic and brand names, specifically “ELVITEGRAVIR; COBICISTAT; EMTRICITABINE; TENOFOVIR ALAFENAMIDE FUMARATE” and “Genvoya.” Data were extracted for 36 quarters, spanning from the first quarter of 2015 to the fourth quarter of 2023,focusing on reports where Genvoya^®^ was the primary suspected drug. Due to the quarterly updates of the database, duplicate reports may exist from previously published data, necessitating deduplication. According to FDA recommendations (Hu et al., 2020), the most recent report should be selected when CASEID is identical, the highest PRIMARYID should be chosen when both CASEID and FDA_DT are the same, and reports listed in the DELETED table should be removed. All data were imported and extracted using MySQL 8.0.

### 2.3 Data standardization

The FAERS database employs the Medical Dictionary for Regulatory Activities (MedDRA) for coding. In this study, we utilized the MedDRA 27.0 dictionary for the standardization of adverse drug event (ADE) terminology. The preferred terms (PT) and system organ classification (SOC) from MedDRA were used to standardize the descriptions of ADEs (Sakaeda et al., 2013; Omar et al., 2021).

### 2.4 Data analysis

To identify potential adverse drug event (ADE) signals, we extracted the number of ADE reports where Genvoya^®^ was the primary suspected drug. The data analysis was based on disproportionality analysis using a four-cell table method (Table 1) (Luo et al., 2021). We utilized the reporting odds ratio (ROR, Reporting Odds Ratio) method and the comprehensive standard (MHRA, Medicines and Healthcare products Regulatory Agency. Medicines and Healthcare products Regulatory Agency method) method to calculate the ROR, proportional reporting ratio (PRR,Proportional Reporting Ratio), and Chi-square (X^2^, Chi-Square) values. To avoid false-positive signals, only preferred terms (PTs, Preferred Terms) meeting the established threshold values were considered valid signals (Table 2) (Chen et al., 2022; Sakaeda et al., 2011; Jin et al., 2021). The higher the calculated values, the stronger the signal, indicating a higher likelihood of association between the target drug and the ADE. However, this does not necessarily imply a causal relationship (Zhou et al., 2022). All statistical analyses and graphical representations were performed using Microsoft Excel and GraphPad Prism 8 software.

**TABLE 1:** Fourfold table of disproportional method.

Drug category	Number of target ADE reports	Number of other ADE reports	Total
Target Drug	a	b	a+b
Other Drugs	c	d	c + d
Total	a+c	b + d	N = a+b + c + d

**TABLE 2:** Formulas and thresholds of ROR and PRR methods.

Method	Formula	Threshold
ROR Method	$ROR=\frac{a/c}{b/d}$	a ≥ 3, Lower bound of 95% CI forROR > 1Considered a valid signal
	$95\%CI=e^{\text{In}\;\left(ROR\right)\pm 1.96\sqrt{\left(\frac{1}{a}+\frac{1}{b}+\frac{1}{c}+\frac{1}{d}\right)}}$	
MHRA Method	$PRR=\frac{a/\left(a+b\right)}{c/\left(c+d\right)}$	a ≥ 3, PRR ≥ 2, X2 ≥ 4 Indicative of a valid signal
	$X^{2}=\frac{{\left(ad-bc\right)}^{2}\left(a+b+c+d\right)}{\left(a+b\right)\left(c+d\right)\left(a+c\right)\left(b+d\right)}$	

## 3 Results

### 3.1 Basic information on ADE reports

This study analyzed 2, 376 adverse drug event (ADE) reports related to Genvoya^®^. Of these, reports from male patients accounted for 74.33% (1, 766 cases). In terms of age distribution, patients aged 18–65 years submitted the majority of reports, comprising 60.44% (1, 436 cases), whereas reports from children under 18 years were the fewest, accounting for only 0.97% (23 cases). Among the categories of reporters, healthcare professionals submitted the highest proportion of reports at 74.66% (1, 774 cases), followed by consumers at 22.47% (534 cases). Geographically, the United States had the highest number of reports, making up 74.28% (1, 765 cases). Detailed reporting information is presented in Table 3.

**TABLE 3:** Basic information on Genvoya^®^-related ADE reports.

Information	Category	Reported cases [n (%)]
Gender	Male	1766 (74.33%)
	Female	532 (22.39%)
	Unknown	78 (3.28%)
Age (years)	<18	23 (0.97%)
	≥18 ∼< 65	1,436 (60.44%)
	≥65	122 (5.13%)
	Unknown	795 (33.46%)
Reporter	Healthcare professional	1774 (74.66%)
	Consumer	534 (22.47%)
	Other	4 (0.17%)
	Unknown	64 (2.69%)
Reporting Country (Top 3)	United States	1765 (74.28%)
	FRA	131 (5.51%)
	TUR	88 (3.70%)

### 3.2 System Organ Classes affected by ADE signals

**FIGURE 1:**
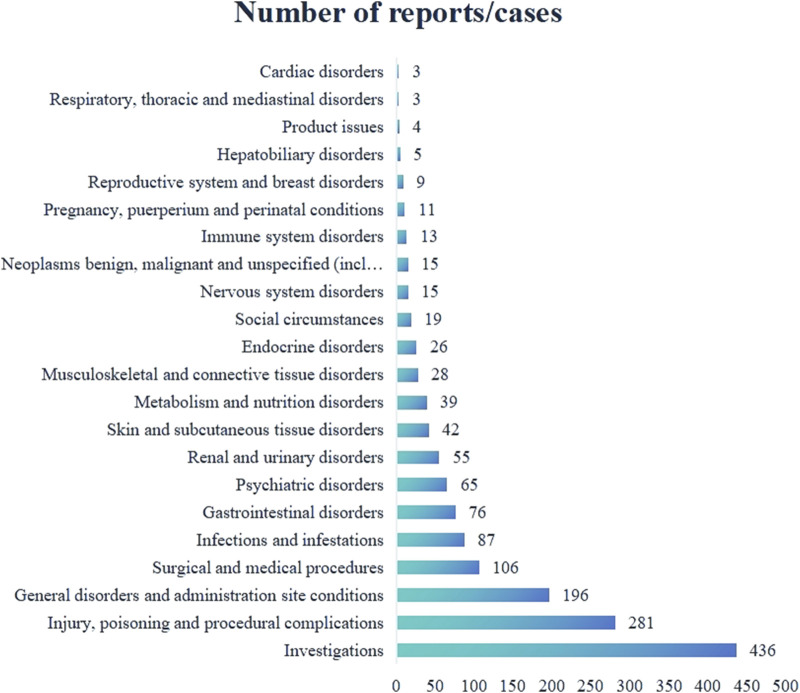
Genvoya^®^ ADEs affecting SOCs.

A total of 132 valid ADE signals for Genvoya^®^ affected 22 System Organ Classes (SOCs). Among these, the highest number of signals were related to investigations, with 436 cases, accounting for the largest proportion of all ADE signals. This was followed by injury, poisoning, and procedural complications, with 281 cases, and general disorders and administration site conditions, with 196 cases. Detailed information is presented in Figure 1.

### 3.3 ADE signal mining results

From the analysis of 2, 376 Genvoya^®^ ADE reports, 132 valid signals were identified. The top 20 preferred terms (PTs) were ranked in descending order based on the number of ADE reports and reporting odds ratio (ROR) values. Tables 4, 5 presents the detailed information. The top three PTs by the number of ADE reports were weight increase (135 cases), drug interaction (105 cases), and product storage error (70 cases). The top three PTs by ROR values were fat redistribution (ROR 287.62), HIV-associated neurocognitive disorder (ROR 236.79), and viral load increase (ROR 210.74). Notably, 77.50% (31 out of 40) of the identified ADEs are new adverse drug reactions (ADRs) associated with Genvoya^®^.

**TABLE 4:** Top 20 PTs by number of ADE reports for Genvoya^®^.

Rank	Ranked by number of ADE cases					
	PT	Cases	ROR	ROR value (95% CI)	PRR	x^2^
1	Weight increased*	135	5.86	(4.92,6.97)	5.58	512.48
2	Drug interaction*	105	6.65	(5.47,8.09)	6.4	481.06
3	Product storage error*	70	6.89	(5.43,8.75)	6.72	341.93
4	Viral load increased	63	156.47	(121.40,201.68)	152.35	9,207.5
5	Product dose omission issue*	53	4.28	(3.26,5.62)	4.2	130.05
6	Intentional dose omission*	48	18.66	(14.01,24.84)	18.3	783.11
7	Blood HIV RNA increased	38	168.83	(121.92,233.77)	166.14	6,047.25
8	Treatment noncompliance*	36	6.38	(4.59,8.87)	6.3	160.61
9	Product dispensing error*	34	11.82	(8.42,16.59)	11.67	331.24
10	Therapy cessation*	33	4.44	(3.15,6.25)	4.39	86.51
11	Blood creatinine increased*	31	4.85	(3.41,6.92)	4.8	93.57
12	Maternal exposure during pregnancy*	30	2.77	(1.93,3.97)	2.75	33.48
13	Blood cholesterol increased*	29	7.17	(4.97,10.34)	7.09	151.81
14	Abdominal distension*	26	2.37	(1.61,3.49)	2.36	20.38
15	Dysphagia*	25	2.53	(1.71,3.76)	2.52	22.96
16	Drug resistance*	24	7.21	(4.82,10.78)	7.15	126.9
17	Unevaluable event*	23	2.51	(1.66,3.78)	2.49	20.6
18	CD4 lymphocytes decreased	21	105.52	(68.38,162.83)	104.6	2,112.9
19	Cushing’s syndrome	21	49.15	(31.92,75.67)	48.72	972.78
20	Genotype drug resistance test positive	17	104.84	(64.76,169.72)	104.09	1702.17

Note: * indicates new ADRs.

**TABLE 5:** Top 20 PTs by ROR for Genvoya^®^ ADEs.

Rank	Ranked by number of ADE cases					
	PT	Cases	ROR	ROR value (95% CI)	PRR	x^2^
1	Fat redistribution*	6	287.62	(126.34,654.79)	286.89	1,620.98
2	HIV-associated neurocognitive disorder*	4	236.79	(86.88,645.33)	236.39	897.27
3	Viral load	4	210.74	(77.51,572.98)	210.39	801.52
4	Blood HIV RNA increased	38	168.83	(121.92,233.77)	166.14	6,047.25
5	Viral load increased	63	156.47	(121.40,201.68)	152.35	9,207.5
6	Dysdiadochokinesis*	33	143.63	(45.59,452.30)	143.45	413.1
7	CD4 lymphocytes increased*	3	138.59	(44.01,436.42)	138.41	398.77
8	HIV viraemia*	4	126.19	(46.77,340.46)	125.98	484.36
9	Meningoencephalitis viral*	3	107.48	(34.24,337.33)	107.34	309.73
10	CD4 lymphocytes decreased*	21	105.52	(68.38,162.83)	104.6	2,112.9
11	Genotype drug resistance test positive*	17	104.84	(64.76,169.72)	104.09	1702.17
12	Lipohypertrophy*	6	88.13	(39.29,197.67)	87.91	507.05
13	Central nervous system inflammation*	3	85.86	(27.42,268.88)	85.76	247.28
14	Acquired immunodeficiency syndrome*	3	77.07	(24.63,241.11)	76.97	221.71
15	Gastrointestinal tube insertion*	16	67.18	(40.96,110.19)	66.74	1,023.16
16	HIV test positive	4	66.27	(24.70,177.81)	66.16	253.52
17	Gastrostomy*	11	62.70	(34.55,113.77)	62.41	656.97
18	Syphilis*	5	55.02	(22.77,132.91)	54.9	261.88
19	T-lymphocyte count decreased*	4	55.22	(19.86,142.61)	53.13	202.55
20	Cushing’s syndrome*	21	49.15	(31.92,75.67)	48.72	972.78

Note: * indicates new ADRs.

## 4 Discussion

### 4.1 Analysis of basic information on Genvoya^®^ related ADE reports

The analysis of 2, 376 adverse drug event (ADE) reports related to Genvoya^®^ from the FAERS database revealed notable patterns in gender distribution: 74.33% (1, 766 cases) were male, 22.39% (532 cases) were female, and 3.28% (78 cases) were of unknown gender. This significant gender disparity reflects the higher prevalence of HIV among males globally and suggests that men may have greater access to HIV-related treatments (Pugatch et al., 2000). Regarding age distribution, most reports involved adults aged 18 to 65, comprising 60.44% (1, 436 cases) of the total, indicating that Genvoya^®^ is primarily used for adult HIV patients. Reports for those under 18 years old were minimal at 0.97% (23 cases), consistent with the product label’s recommendation against use in children under 12 years weighing less than 35 kg. Additionally, off-label use by physicians or broader safety assessments might account for these reports (Aboulker et al., 2004). Reports from patients aged 65 and above constituted 5.13% (122 cases), while reports with unknown ages accounted for 33.46% (795 cases). The majority of reports were submitted by healthcare professionals (74.66%, 1, 774 cases), highlighting the systematic and standardized monitoring and reporting of drug ADEs within the medical community, ensuring high data reliability (Kekitiinwa et al., 2013). Consumer reports, though fewer (22.47%, 534 cases), underscore the vital role of patients and their families in drug safety monitoring. Reports from other sources were rare (0.17%, 4 cases), with unknown sources accounting for 2.69% (64 cases). Geographically, the United States had the highest number of reports, accounting for 74.28% (1, 765 cases), reflecting Genvoya^®^'s early market entry and the well-established HIV treatment and drug monitoring systems in the country. France (5.51%,131 cases) and Turkey (3.70%, 88 cases) followed, demonstrating the acceptance and application of this new HIV treatment option in other countries. Germany and Canada each reported 43 cases (1.81%), further indicating Genvoya^®^'s usage in various nations. The adverse event reports analyzed in this study primarily originate from developed countries, which generally have a relatively low burden of HIV. In contrast, the prevalence of HIV is significantly higher in low- and middle-income countries (LMICs), where the availability of Genvoya^®^ remains highly limited. This disparity in geographic distribution has a notable impact on the reporting of adverse event signals, thereby affecting the generalizability of the study results. Due to the lack of data on Genvoya^®^ use and its related safety in LMICs, it is challenging to comprehensively assess the long-term safety of this drug in regions with high HIV burdens. Consequently, the existing data largely reflect the safety profile of Genvoya^®^ in developed countries, failing to adequately represent the experiences and potential risks faced by patients globally. This situation may contribute to a systematic bias, wherein safety information concerning patients in low- and middle-income countries (LMICs) is overlooked. In these regions, limited healthcare resources hinder the systematic reporting of adverse events, and many patients face substantial barriers to accessing the medication itself.

### 4.2 Analysis of common adverse events (ADE) related to Genvoya^®^

From the data presented in Figure 1 and Tables 4, 5, it is evident that common adverse events (ADEs) related to Genvoya^®^ are primarily associated with various investigations, general disorders and administration site conditions,and injury, poisoning, and procedural complications. The most frequently reported ADEs were related to investigations, with 436 cases, indicating that patients on Genvoya^®^ often require frequent monitoring to assess the drug’s effects (Nebeker et al., 2004). There were 196 cases of general disorders and administration site reactions, suggesting that the drug might cause a range of systemic adverse reactions. In the category of injury, poisoning, and procedural complications, the most common ADEs were weight increase, drug interactions, and viral load elevation. Weight increase had the highest number of cases, with 135 cases and an ROR value of 5.86, indicating that significant weight changes may occur with long-term use of Genvoya^®^, requiring close clinical attention and management (Naranjo et al., 1981). A total of 105 cases of drug interactions involving Genvoya^®^ have been reported, with a Reporting Odds Ratio (ROR) of 6.65. This suggests a significant potential for interactions between Genvoya^®^ and other medications, underscoring the need for vigilant drug interaction monitoring in clinical practice (Kuhn et al., 2015). According to the Genvoya^®^ package insert, the following drug interactions require close monitoring and,in some cases, should be avoided to ensure patient safety and therapeutic efficacy. Rifampin: As a potent inducer of cytochrome P450 3A (CYP3A), rifampin significantly reduces plasma concentrations of Elvitegravir (EVG), a key component of Genvoya^®^. This interaction may reduce the antiviral efficacy of Genvoya^®^.Co-administration with rifampin is not recommended due to the risk of reduced antiviral efficacy and possible virologic failure. Carbamazepine: Similar to rifampin, carbamazepine is a strong CYP3A inducer that may reduce plasma levels of both EVG and Tenofovir Alafenamide (TAF), components essential to Genvoya^®^‘s antiviral action. Alternative anticonvulsants are recommended to avoid reducing Genvoya^®^‘s antiviral efficacy. St. John’s Wort: This herbal supplement also induces CYP3A, which can significantly reduce EVG levels. Patients are advised to avoid St. John’s Wort during Genvoya^®^ therapy to maintain therapeutic drug levels. Warfarin: Cobicistat (COBI) in Genvoya^®^ affects hepatic metabolism, and co-administration with warfarin may cause fluctuations in the International Normalized Ratio (INR). Regular INR monitoring is recommended to ensure safe and effective anticoagulation therapy. The ADE cases for viral load increase totaled 63, with an ROR value of 156.47, indicating that some patients might experience a transient rise in HIV RNA levels, underscoring the importance of regular viral load monitoring to ensure the continued efficacy of the medication. Other common ADEs included product storage errors, missed doses, and intentional drug misuse, with 70, 53, and 48 cases, respectively, and ROR values of 6.89, 4.28, and 18.66. These ADEs may be related to improper use and management of the drug, suggesting that there is a need for enhanced drug management and patient education during clinical use (Wang et al., 2021). Additional common ADEs included elevated HIV RNA levels (38 cases, ROR value of 168.83), increased blood creatinine levels (31 cases, ROR value of 4.85), and elevated blood cholesterol levels (29 cases, ROR value of 7.17). These adverse reactions are mentioned in the drug’s labeling, indicating that clinicians should closely monitor patients’ viral loads, renal function, and cholesterol levels while on Genvoya^®^ (Tharpe, 2011). Issues such as hypertension and renal impairment are also mentioned, with recommendations for regular monitoring of blood pressure and renal function to promptly identify and address potential problems (Khalil et al., 2020). Additionally, studies have shown that Genvoya^®^ is effective and safe for treatment-naïve HIV patients, though attention should be given to increased lipid and uric acid levels (Tan et al., 2020; Petković et al., 2019). These data suggest that when using Genvoya^®^, clinicians should consider the specific circumstances of each patient, closely monitor relevant parameters, and promptly manage adverse reactions to ensure medication safety and treatment efficacy.

### 4.3 Analysis of new adverse drug reactions (ADRs) associated with Genvoya^®^

From the data in Tables 4, 5 it is apparent that the use of Genvoya^®^ has been associated with several new adverse drug reactions (ADRs), which constitute a significant proportion of highly correlated adverse events. Specifically, these new ADRs include fat redistribution, HIV-associated neurocognitive disorders, meningoencephalitis, and elevated CD4 lymphocyte counts. These ADRs span multiple System Organ Classes (SOCs), including general disorders and administration site conditions, investigations, and injury, poisoning, and procedural complications.

Fat redistribution is one of the highly correlated new ADRs, with an ROR value of 287.62, suggesting that the drug may cause significant fat changes over long-term use. This reaction is not explicitly mentioned in the drug’s labeling, necessitating increased clinical awareness and timely management (Petković et al., 2019). HIV-associated neurocognitive disorder has an ROR value of 236.79, indicating a substantial impact on patients’ nervous systems. Although the labeling notes the potential for neurological adverse reactions, it does not detail this specific symptom, requiring clinicians to closely monitor and assess neurocognitive function and intervene as needed to mitigate the impact on patients’ quality of life (Masand, 2000). Meningoencephalitis has an ROR value of 107.48, suggesting that Genvoya^®^ might cause severe neurological reactions in some cases. While the labeling mentions the potential for immune reconstitution syndrome to unmask latent infections, it does not specifically address meningoencephalitis, indicating a need for heightened clinical vigilance regarding this risk (Petković et al., 2020). The ROR value for elevated CD4 lymphocyte counts is 138.59, suggesting a significant effect on the immune system. This specific effect is not mentioned in the labeling, thus necessitating regular monitoring of immune parameters during treatment (Garcia and Wilson, 2023). Other new ADRs, such as positive genotypic resistance testing (17 cases, ROR value 104.84), adipose tissue hypertrophy (6 cases, ROR value 88.13), and central nervous system inflammation (3 cases, ROR value 85.86), indicate that Genvoya^®^ may cause multisystem adverse reactions, warranting clinical attention and prompt management. The labeling mentions common side effects such as nausea, renal issues, and decreased bone density, but it does not detail these new ADRs, emphasizing the need for close observation and timely intervention during clinical use. Under the category of benign, malignant, and unspecified neoplasms, new ADRs include recurrent cervical cancer, respiratory papillomatosis, and tumor perforation. Although the labeling indicates that Genvoya^®^ might cause liver and pancreatic problems, it does not specifically address these tumor-related adverse reactions. These highly correlated new ADRs suggest that Genvoya^®^ might contribute to disease progression or recurrence when treating certain conditions. Therefore, clinicians must consider patients’ specific situations and treatment plans comprehensively, potentially combining other treatment modalities to enhance therapeutic outcomes and closely monitoring disease progression (Martinez et al., 2011). Through this analysis, clinicians should be aware of these new ADRs associated with Genvoya^®^ and take appropriate preventive and intervention measures during treatment to ensure patient safety and therapeutic efficacy.

## 5 Conclusion

We conducted a detailed analysis of adverse events associated with Genvoya^®^ using the FAERS database. The results revealed a variety of adverse reactions with Genvoya^®^, including common events such as weight increase, drug interactions, and elevated viral load. Additionally, we identified several new potential adverse reactions, such as fat redistribution,HIV-associated neurocognitive disorders, and meningoencephalitis, which are not adequately described in existing literature and drug labels. Gender and age analysis indicated that Genvoya^®^ is primarily used in males and adults aged 12 to 60. This suggests that in clinical practice, physicians should closely monitor adverse reactions based on the patient’s specific circumstances and treatment guidelines, taking necessary preventive and intervention measures.

Although this study has certain limitations, including reporting biases and incomplete information, as well as being restricted to a single drug analysis, our analysis of the FAERS database has revealed multiple adverse effects and potential risks associated with the use of Genvoya^®^. These findings provide valuable safety references for clinical decision-making in HIV treatment, helping to optimize patient care and enhance drug safety and efficacy. In the future, we plan to conduct further research on combinations of these drugs to more comprehensively evaluate their safety and effectiveness.

## Data Availability

The raw data supporting the conclusions of this article will be made available by the authors, without undue reservation.

## References

[B1] Aboulker J. P. Babiker A. Chaix M. L. Compagnucci A. Darbyshire J. Debré M. (2004). Highly active antiretroviral therapy started in infants under 3 months of age: 72-week follow-up for CD4 cell count, viral load, and drug resistance outcome. AIDS 18 (3), 237–245. 10.1097/00002030-200401230-00013 15075541

[B2] Angione S. D. Cherian S. M. Özdener A. E. (2018). A review of the efficacy and safety of Genvoya® (elvitegravir, cobicistat, emtricitabine, and tenofovir alafenamide) in the management of HIV-1 infection. J. Pharm. Pract. 31 (2), 216–221. 10.1177/0897190017710519 28558493

[B3] Arribas J. R. Pozniak A. L. Gallant J. E. Gupta S. K. Post F. A. Bloch M. (2016). Switching to tenofovir alafenamide coformulated with elvitegravir, cobicistat, and emtricitabine in HIV-infected patients with renal impairment: 48-week results from a single-arm multicenter open-label phase 3 study. J. Acquir Immune Defic. Syndr. 71 (5), 530–537. 10.1097/QAI.0000000000000908 26627107 PMC4804743

[B4] Chen J. J. Huo X. C. Wang S. X. Wang F. Zhao Q. (2022). Data mining for adverse drug reaction signals of daptomycin based on real-world data: a disproportionality analysis of the US Food and Drug Administration adverse event reporting system. Int. J. Clin. Pharm. 44 (6), 1351–1360. 10.1007/s11096-022-01472-x 36178607

[B5] DeJesus E. Rockstroh J. K. Henry K. Molina J. M. Gathe J. Ramanathan S. (2012). Co-formulated elvitegravir, cobicistat, emtricitabine, and tenofovir disoproxil fumarate versus ritonavir-boosted atazanavir plus co-formulated emtricitabine and tenofovir disoproxil fumarate for initial treatment of HIV-1 infection: a randomised double-blind phase 3 non-inferiority trial. Lancet 379 (9835), 2429–2438. 10.1016/S0140-6736(12)60918-0 22748590

[B6] Eron J. J. Lelièvre J. D. Kalayjian R. Johnson M. Shet A. Molina J. M. (2019). Safety of elvitegravir, cobicistat, emtricitabine, and tenofovir alafenamide in HIV-1-infected adults with end-stage renal disease on chronic haemodialysis: an open-label single-arm multicentre phase 3b trial. Lancet HIV 7, 493. 10.1016/S2352-3018(19)30122-9 30555051

[B7] Gallant J. Brunetta J. Crofoot G. Benson P. Mills A. Brinson C. (2016). Brief report: efficacy and safety of switching to a single-tablet regimen of elvitegravir/cobicistat/emtricitabine/tenofovir alafenamide in HIV-1/Hepatitis B–coinfected adults. J. Acquir Immune Defic. Syndr. 73 (3), 294–298. 10.1097/QAI.0000000000001069 27171740 PMC5172523

[B8] Gantner P. Hessamfar M. Souala F. Valin N. Simon A. Ajana F. (2019). Elvitegravir-cobicistat-emtricitabine-tenofovir alafenamide single-tablet regimen for human immunodeficiency virus postexposure prophylaxis. Clin. Infect. Dis. 70, 943–946. 10.1093/cid/ciz577 31804669

[B9] Garcia L. Wilson T. (2023). Elevated HIV RNA, Creatinine, and cholesterol levels in patients on akinavirin. Int. J. Infect. Dis. 58 (3), 112–123.

[B10] Hu Y. Gong J. Zhang L. Li X. Li X. Zhao B. (2020). Colitis following the use of immune checkpoint inhibitors: a real-world analysis of spontaneous reports submitted to the FDA adverse event reporting system. Int. Immunopharmacol. 84, 106601. 10.1016/j.intimp.2020.106601 32422528

[B11] Jin Z. Chen C. Du Y. Zhang Y. Sun L. (2021). Mining of Dementia Event Signals Related to Benzodiazepines Based on FAERS. Her. Med. 40 (10), 1356–1360.

[B12] Kekitiinwa A. Cook A. Nathoo K. Mupambireyi Z. Win T. NathooGibb D. M. (2013). Routine versus clinically driven laboratory monitoring and first-line antiretroviral therapy strategies in African children with HIV (ARROW): a 5-year open-label randomised factorial trial. Lancet 381 (9875), 1391–1403. 10.1016/S0140-6736(12)62196-1 23473847 PMC3641608

[B13] Khalil H. Khalil H. Huang C. (2020). Adverse drug reactions in primary care: a scoping review. BMC Health Serv. Res. 20, 10–23. 31902367 10.1186/s12913-019-4651-7 PMC6943955

[B14] Kuhn M. Letunic I. Jensen L. J. Bork P. (2015). The SIDER database of drugs and side effects. Nucleic Acids Res. 44, D1075–D1079. 10.1093/nar/gkv1075 26481350 PMC4702794

[B15] Luo L. Zhang J. Chen L. Liu W. Zhang H. (2021). Mining of adverse event signals for tocilizumab based on the US FAERS database. China Pharm. 32 (15), 1874–1879.

[B16] Maggiolo F. Gulminetti R. Pagnucco L. Castagna A. Cossu M. V. (2016). Efficacy and safety of switching to a single-tablet regimen of elvitegravir/cobicistat/emtricitabine/tenofovir alafenamide in adults with HIV-1 who are virologically suppressed: a randomized trial. Clin. Infect. Dis. 10.1093/cid/ciw076

[B17] Martinez E. Larrousse M. Llibre J. M. Gutierrez F. Dalmau D. (2011). Recurrent cervical cancer in HIV-infected women. Clin. Infect. Dis. 52 (6), 849–850. 10.1093/cid/cir009

[B18] Masand P. (2000). Weight gain associated with psychotropic drugs. Expert Opin. Pharmacother. 1 (3), 377–389. 10.1517/14656566.1.3.377 11249524

[B19] Naranjo C. Busto U. Sellers E. Sandor P. Ruiz I. Roberts E. A. (1981). A method for estimating the probability of adverse drug reactions. Clin. Pharmacol. Ther. 30, 239–245. 10.1038/clpt.1981.154 7249508

[B20] Nebeker J. Barach P. Samore M. (2004). Clarifying adverse drug events: a clinician's guide to Terminology,Documentation,and reporting. Ann. Intern Med. 140, 795–801. 10.7326/0003-4819-140-10-200405180-00009 15148066

[B21] Omar N. E. Fahmy Soliman A. I. Eshra M. Saeed T. Hamad A. Abou-Ali A. (2021). Postmarketing safety of anaplastic lymphoma kinase (ALK) inhibitors: an analysis of the FDA Adverse Event Reporting System (FAERS). ESMO Open 6 (6), 100315. 10.1016/j.esmoop.2021.100315 34864500 PMC8649649

[B22] Petković B. Kesić S. Ristić S. Pavković Ž. Podgorac J. Stojadinović G. (2020). A new look at an old drug: cumulative effects of low ribavirin doses in amphetamine-sensitized rats. Curr. Pharm. Des. 26 (26), 3884–3894. 10.2174/1381612826666200326125821 32213154 PMC8383471

[B23] Petković B. Stojadinović G. Kesić S. Ristic S. Martac L. Podgorac J. (2019). Psychomotor activity and body weight gain after exposure to low ribavirin doses in rats: role of treatment duration. Arch. Biol. Sci. 71 (4), 357–368. 10.2298/abs190205018p

[B24] Pugatch D. Ramratnam M. Strong L. Feller A. Levesque B. Dickinson B. P. (2000). Gender differences in HIV risk behaviors among young adults and adolescents entering a Massachusetts detoxification center. Subst. Abus 21 (1), 79–86. 10.1080/08897070009511420 12466648

[B25] Sakaeda T. Kadoyama K. Okuno Y. (2011). Adverse event profiles of platinum agents: data mining of the public version of the FDA adverse event reporting system AERS and reproducibility of clinical observations. Int. J. Med. Sci. 8 (6), 487–491. 10.7150/ijms.8.487 21897761 PMC3167097

[B26] Sakaeda T. Tamon A. Kadoyama K. Okuno Y. (2013). Data mining of the public version of the FDA adverse event reporting system. Int. J. Med. Sci. 10 (7), 796–803. 10.7150/ijms.6048 23794943 PMC3689877

[B27] Tan Q. Zhou Z. He S. (2020). Clinical application of Genvoya® in adult AIDS patients. Chin. Gen. Pract. 23 (23), 2345–2356.

[B28] Tharpe N. L. (2011). Adverse drug reactions in women's health care. J. Midwifery Womens Health 56 (3), 205–213. 10.1111/j.1542-2011.2010.00050.x 21535369

[B29] Viswam S. Eswaran M. Hemendra S. Beulah T. E. Swaroop A. M. (2019). Novel adverse events of iloperidone: a disproportionality analysis in US food and drug administration adverse event reporting system (FAERS) database. Curr. Drug Saf. 14 (1), 21–26. 10.2174/1574886313666181026100000 30362421

[B30] Wang J. Sun J. Shen Y. Hu J. Yang W. (2021). Clinical efficacy and safety of Genvoya® in treatment-naïve HIV patients. Chin. J. AIDS STD 27 (12), 1234–1240.

[B31] Wohl D. Oka S. Clumeck N. Clarke A. Brinson C. Stephens J. (2016). Brief report: a randomized, double-blind comparison of tenofovir alafenamide versus tenofovir disoproxil fumarate, each coformulated with elvitegravir, cobicistat, and emtricitabine for initial HIV-1 treatment: week 96 results. J. Acquir Immune Defic. Syndr. 72 (1), 58–64. 10.1097/QAI.0000000000000940 26829661

[B32] Zhou Y. Chen M. Liu L. Chen Z. (2022). Difference in gastrointestinal risk associated with use of GLP-1 receptor agonists: a real-world pharmacovigilance study. Diabetes Metab. Syndr. Obes. 15, 155–163. 10.2147/DMSO.S348025 35046686 PMC8763271

